# Ovarian Aging: The Silent Catalyst of Age-Related Disorders in Female Body

**DOI:** 10.14336/AD.2024.1468

**Published:** 2026-01-27

**Authors:** Xingyu Liu, Yuanqu Zhao, Yanzhi Feng, Shixuan Wang, Aiyue Luo, Jinjin Zhang

**Affiliations:** ^1^Department of Obstetrics and Gynecology, Tongji Hospital, Tongji Medical College, Huazhong University of Science and Technology, Wuhan, 430030, China.; ^2^National Clinical Research Center for Obstetrical and Gynecological Diseases, Huazhong University of Science and Technology, Wuhan, 430030, China.; ^3^Key Laboratory of Cancer Invasion and Metastasis, Ministry of Education, Huazhong University of Science and Technology, Wuhan, 430030, China.

**Keywords:** Ovarian aging, menopause, age-related diseases, anti-aging therapy

## Abstract

Age-related diseases have emerged as a global concern as the population ages. Consequently, understanding the underlying causes of aging and exploring potential anti-aging interventions is imperative. In females, the ovaries serve as the principal organs responsible for ovulation and the production of female hormones. The aging ovaries are related to infertility, menopause, and associated menopausal syndromes, with menopause representing the culmination of ovarian aging. Current evidence indicates that ovarian aging may contribute to dysfunction across multiple organ systems, including, but not limited to, cognitive impairment, osteoporosis, and cardiovascular disease. Nevertheless, due to the widespread distribution of sex hormone receptors throughout the body, ovarian aging affects not only these specific organs but also influences a broader spectrum of age-related diseases in women. Despite this, the impact of ovarian aging on overall age-related diseases has been largely neglected. This review provides a thorough summary of the impact of ovarian aging on age-related diseases, encompassing the nervous, circulatory, locomotor, urinary, digestive, respiratory, and endocrine systems. Additionally, we have outlined prospective therapeutic approaches for addressing both ovarian aging and age-related diseases, with the aim of mitigating their impacts and preserving women's fertility, physical health, and psychological well-being.

## 1. Introduction

The process of aging is a universal experience that all individuals undergo. Aging drives the onset and progression of multiple organ disorders, including cardiovascular and cerebrovascular disease, diabetes mellitus, and cancer [1]. By 2050, the global population aged 65 and older is predicted to exceed 2 billion, up from 617 million today, thereby accounting for about 20% of the overall population [2]. The global trend of population aging has spurred extensive research into age-related diseases, which is crucial for achieving healthy aging. Investigating the factors underlying aging and age-related diseases is crucial for developing prevention and treatment strategies.

**Figure 1.**
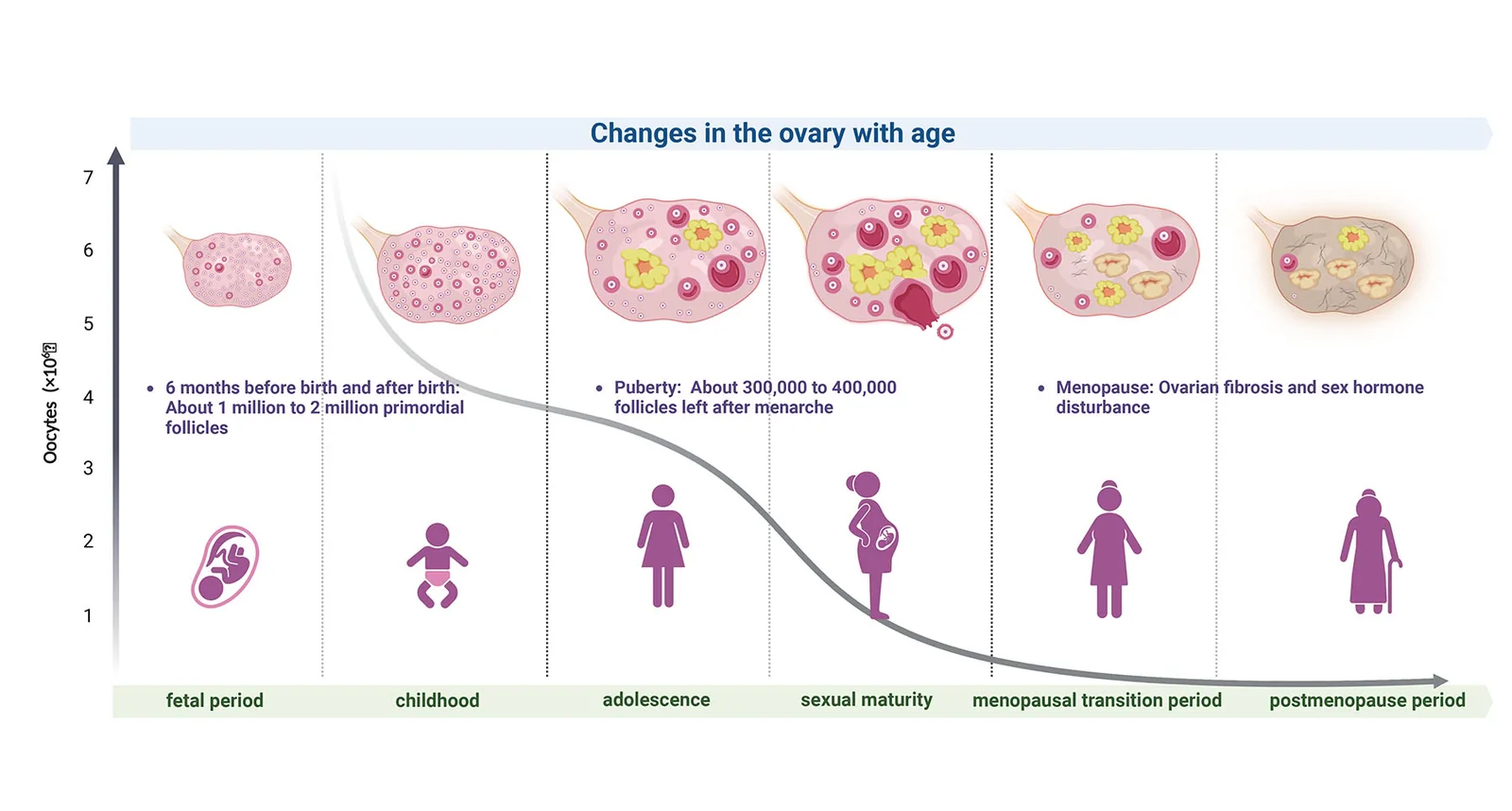
**The life stages of the ovary and female body**. Created with BioRender.com.

In humans, the ovaries age earlier than other organs [3], governing the female reproductive lifespan and sex hormone production. Ovarian aging is a natural process characterized by a decrease in both follicle quantity and quality, fluctuations in hormone levels, and a heightened risk of age-related diseases, ultimately resulting in infertility and menopause as primary outcomes (Fig.1) [4]. The number of primordial follicles peaks during gestation and declines nonlinearly over time, leaving fewer than 1,000 by menopause, which typically occurs around 51 years of age [5]. During perimenopause, the decline in ovarian follicles lowers inhibin-B, prompting the anterior pituitary to produce follicle-stimulating hormone (FSH) to boost estradiol production in ovaries. However, as ovarian function declines, FSH levels keep rising, but estradiol production falters, resulting in persistently high FSH levels [6].

Given the wide distribution of estrogen receptors (ERs) in various organs and tissues, the ovaries are crucial for maintaining bodily functions beyond the reproductive system [7]. For example, estrogen is crucial for many physiological activities, including improving endothelium-dependent vascular function and anti-atherosclerotic activity, suppressing immune activation and neuroinflammation by microglia and astrocytes in the brain, and supporting muscle maintenance and resilience [8]. Numerous studies have demonstrated that ovarian aging significantly impacts the structure and function of the heart, bone, and brain through estrogen signaling, thereby elevating the risk of cardiovascular disease, osteoporosis, and cognitive impairment [3, 4, 9]. Apart from these, ovarian aging may contribute to obesity [10], skin aging [11], chronic kidney disease [12], hepatic aging [13], type 2 diabetes mellitus [14], chronic obstructive pulmonary disease [15], etc. Notably, premature ovarian aging has been reported to reduce overall life expectancy [3, 9]. Consequently, ovarian aging may be one of the key factors for systemic age-related diseases, and its impact on the overall organ systems should not be neglected.

To address ovarian aging and its related diseases, it is crucial to identify the etiological and risk factors contributing to this process. Genetic variants, such as FIGLA, SOHLH1, LHX8, and NR5A1, are associated with accelerated ovarian aging [16]. Furthermore, lifestyles including intense physical activity, significant weight loss, smoking, vegetarian or high-carbohydrate diets, and excessive intake of polyunsaturated fats may exacerbate ovarian aging [17]. Moreover, natural and artificial endocrine disrupting chemicals exposure through contaminated food, water, air, dermal contact (e.g., cosmetics), and occupational exposure (e.g., pesticides) can disrupt the endocrine system, thereby causing ovarian aging [18]. Thus, gene therapy, medications, lifestyle modifications and environmental improvements may effectively slow the process of ovarian aging.

This paper seeks to offer a systematic and comprehensive overview of the impacts of ovarian aging on age-related diseases across various organ systems, thereby advancing the understanding of the interrelationship between ovarian aging and age-related diseases. For clarity in the discussion of this topic, we have categorized the body into ten systems: the nervous, circulatory, motor, urinary, digestive, respiratory, endocrine, integumentary, immune, and reproductive systems. Furthermore, therapeutic interventions targeting both ovarian aging and age-related diseases have been discussed, aiming to facilitate the clinical translation of anti-aging approaches.

## 2. Ovarian aging and age-related diseases

### 2.1 Motor system

#### 2.1.1 Osteoporosis

Osteoporosis, a common bone metabolism-related disorder, is closely linked to aging and characterized by an imbalance between osteoblast-mediated bone formation and osteoclast-mediated bone resorption, leading to the disruption of bone homeostasis [19]. The incidence of osteoporosis is notably higher in women than in men, largely due to the pivotal role of estrogen in regulating bone metabolism [20]. Approximately 30% of cortical bone and 50% of trabecular bone are lost throughout a woman’s lifespan, with menopause accounting for a substantial portion of this bone loss [21]. Iqbal *et al.* have demonstrated that nearly half of a woman's total bone mass loss occurs within the first five years post-menopause [22]. The most pronounced bone loss typically begins two years prior to the final menstrual period, during which the levels of estrogen remain normal but that of FSH are elevated [22].

Estrogen primarily influences bone cells via ERα, activating the ER signaling pathway to promote osteoblast differentiation and inhibit osteoclast activity [23]. Melville *et al*. found that mice with osteoblast-specific ERα knocked out exhibited lower bone mass and strength [24]. The proliferation ability and collagen gene expression of cultured rat osteoblast-like cells were significantly enhanced after 17β-estradiol treatment [25]. In addition, estrogen influences the RANKL/osteoprotegerin system [26]. The receptor activator of nuclear factor -κ B ligand (RANKL), a member of the TNF ligand family, has been identified as an essential cytokine for osteoclast formation and activation [26]. The addition of RNALK to cultured bone marrow macrophages (BMMs) in vitro led to the formation of osteoclasts [27]. When estrogen is deficient, the abundance of at least five cytokines increases, including interleukin-1 (IL-1), interleukin-6 (IL-6), tumor necrosis factor (TNF), macrophage colony-stimulating factor (M-CSF), granulocyte-macrophage colony-stimulating factor (GM-CSF), with IL-1 and TNF being the most potent activators of bone resorption and inhibitors of bone formation [26, 28]. TNF activates PI3K/protein kinase B (Akt) signaling, thereby promoting RANKL-induced osteoclast formation [29], while IL-1 exerts a synergistic effect on this [30]. Furthermore, estrogen has been shown to reduce the expression and secretion of lysosomal enzymes, with 17β-estradiol treatment leading to a significant decrease in lysosomal secretion from osteoclasts [31]. Additionally, 17β-estradiol promoted apoptosis of osteoclasts in vitro and in vivo, inducing apoptosis two- to threefold more effectively than in untreated controls [32].

Beyond its direct effects on bone cells, estrogen can influence bone metabolism through the regulation of other hormones. It increases the expression of parathyroid hormone and calcitonin in parathyroid and C cells, which helps prevent osteoporosis by regulating bone metabolism [33]. Furthermore, estrogen may regulate bone metabolism indirectly by modulating gut microbiota, intestinal calcium absorption, and renal calcium handling [34, 35]. In addition, estrogen reduction caused by ovarian aging leads to increased FSH levels. Sun *et al*. found that FSHβ and FSH receptor (FSHR) gene knockout mice had no bone loss despite hypogonadism, suggesting that FSH promotes the development of osteoporosis [36].

**Figure 2.**
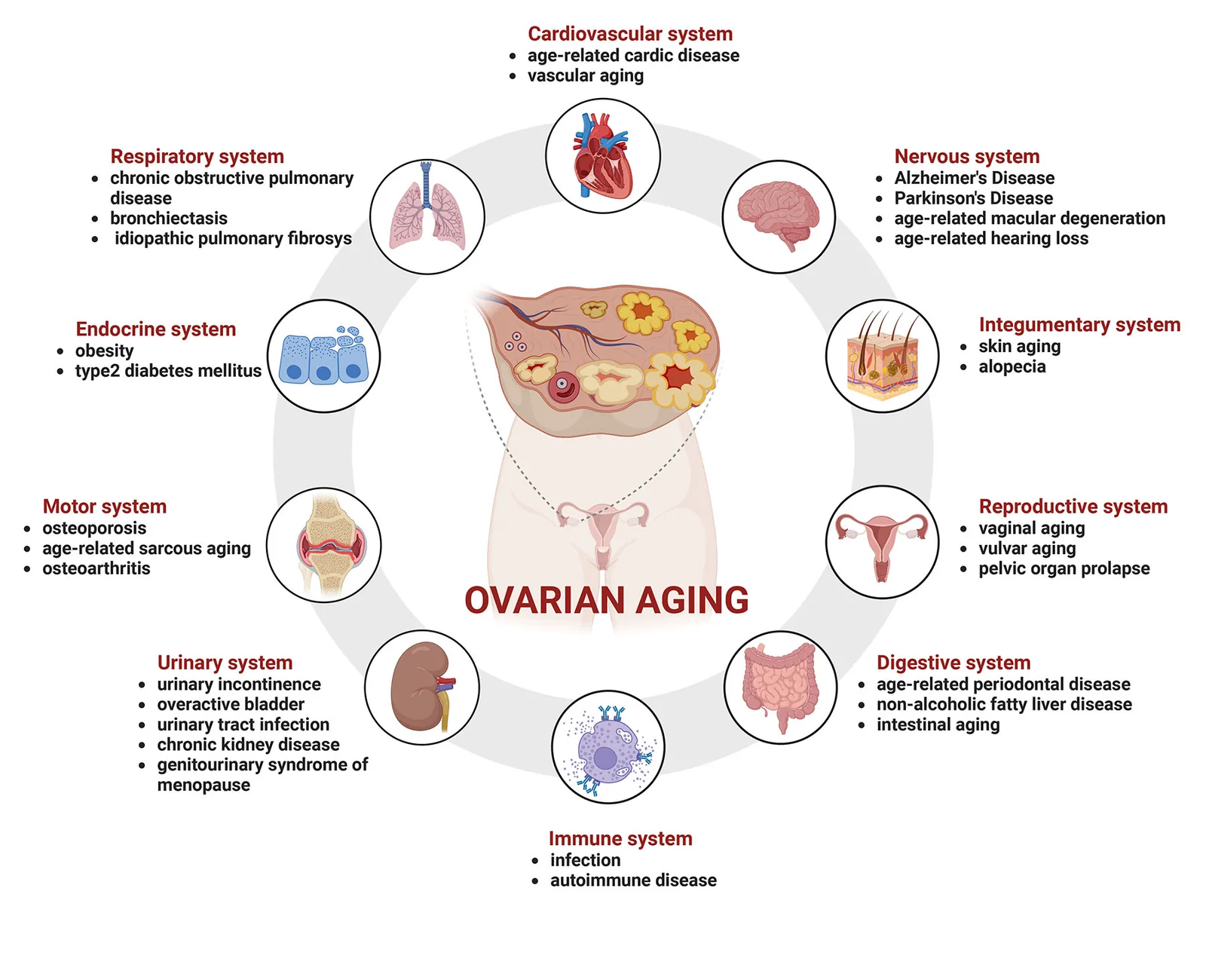
**The potential impact of ovarian aging on age-related diseases in multiple systems and organs**. Created with BioRender.com.

In summary, the influence of estrogen on bone metabolism is multi-channel and dimensional. Notably, estrogen-progestin therapy may be more effective than estrogen alone in enhancing spinal bone mineral density (BMD) in women with postmenopausal osteoporosis (Fig. 2) [37, 38].

#### 2.1.2 Age-related sarcous aging

In addition to bone metabolism, changes in hormone levels caused by ovarian aging affect muscle. Postmenopausal muscle aging is primarily characterized by a reduction in muscle mass, muscle atrophy, and decreased muscle strength. Mechanisms associated with muscle atrophy and muscle mass loss due to estrogen deficiency include protein turnover, muscle cell apoptosis, and ubiquitin protease degradation [39]. Decreased estrogen levels also lead to elevated concentrations of cytokines such as IL-6 and TNF, which exacerbate muscle deterioration [28]. Elevated IL-6 levels are associated with a reduction in insulin-like growth factor 1 (IGF-1), a key regulator of muscle growth and maintenance [40]. Additionally, TNF-α has been shown to promote muscle cell apoptosis, further contributing to muscle atrophy [40]. Muscle loss contributes to decreased muscle strength, and estrogen also affects the contractility of skeletal muscle by regulating myosin [41]. Moreover, estrogen plays an essential role in the maintenance and function of satellite cells, which are critical for muscle regeneration [42]. Navira *et al.* demonstrated that estrogen replacement therapy can increase appendicular lean mass in patients with premature ovarian insufficiency (POI) [43]. However, a systematic meta-analysis by Ayesha *et al*. found no clear beneficial or harmful relationship between estrogen supplementation and muscle mass [44]. In conclusion, the mechanisms by which estrogen influences muscle aging require further clarification, and the potential application of estrogen in treating muscle loss and frailty due to ovarian aging remains to be explored.

#### 2.1.3 Osteoarthritis

Statistically, women suffer from osteoarthritis and related pain at twice the rate of men. This disparity becomes more pronounced during menopause, with over 50% of women experiencing osteoarthritis or arthralgias, potentially due to changes in estrogen levels caused by ovarian aging [45, 46]. ERs found in bone, ligaments, synovium, and cartilage indicate that varying estrogen levels may affect joint function [45, 47, 48]. Progesterone may impact joint function indirectly by promoting ERα expression and affecting chondrocyte survival [48]. Current evidence implicates that estrogen deficiency may play a significant role in triggering inflammatory responses, oxidative stress, and the heightened production of inflammatory cytokines, all of which may contribute to the progression of osteoarthritis [46]. Some studies have reported a protective effect of estrogen replacement therapy on cartilage and joint health, while others have found no significant therapeutic benefit [49]. The pathogenesis of osteoarthritis associated with decreased estrogen requires further investigation to identify more effective treatment strategies.

### 2.2 Urinary system

#### 2.2.1 Urinary incontinence (UI) and overactive bladder (OAB)

ERs are widely expressed in the urethra, vagina, bladder trigone, pubococcygeal muscle, pelvic floor muscle, and anal levator interstitial tissue. These receptors play pivotal roles in increasing muscle fiber density, modulating urinary tract responses to adrenergic stimulation, and regulating collagen synthesis and metabolism in the lower reproductive tract [50-52]. Estrogen may inhibit Rho-kinase activity, thereby reducing calcium sensitization and attenuating bladder smooth muscle contraction, potentially explaining the higher incidence of overactive bladder (OAB) observed after menopause [53]. Decreased estrogen levels can reduce acetylcholine release from nerve fibers, further impairing detrusor contraction ability [51]. Overall, the decline in circulating estrogen during menopause results in a loss of dermal collagen in the dense connective tissue of the bladder and urethra, adversely affecting their contractile functions. This reduction in estrogen also hampers the proliferation of smooth muscle cells, decreases blood vessel density, and induces the conversion of urogenital fibroblasts into senescent cells [52]. Collectively, these changes result in structural and functional deterioration, manifesting as frequent urination and incontinence. Estrogen supplementation has been shown to enrich the loose, vascularized connective tissue of the urethra, enhance the sympathetic nerve distribution within pelvic floor tissues, and improve UI [54, 55]. The method of estrogen administration seems to be a key factor. A meta-analysis found that while topical estrogen therapy improves UI, systemic therapy with combined equine estrogen may exacerbate it [56]. Furthermore, combined estrogen and progestin were likely to negatively impact UI improvement [57]. The type, method, and dosage of estrogen require further exploration to ascertain their efficacy in alleviating UI.

Progesterone receptor expression is inconsistently reported across the bladder, trigone, and vagina, suggesting that progesterone may influence OAB symptoms. This could explain the heightened prevalence of lower urinary tract symptoms during menstruation and pregnancy [58-60].

In addition to UI, OAB is closely related to aging, which includes a series of symptoms such as frequent urination, urgent urination, and incontinence. Local estrogen therapy can effectively relieve these lower urinary tract symptoms that occur after menopause [61].

#### 2.2.2 Urinary tract infection (UTI)

The prevalence of urinary tract infections (UTIs) in women increases with age, affecting approximately 8-10% of postmenopausal women, of whom 5% experience recurrent urinary tract infections (rUTIs) [62]. The initial stages of uncomplicated UTIs in women are often marked by the contamination of the periurethral and vaginal regions with pathogens originating primarily from the intestinal flora [63]. A study involving vaginal cultures from 140 women demonstrated significantly higher rates of vaginal *Escherichia coli* colonization in individuals with recurrent UTIs compared to those without UTIs [64]. The decline in estrogen levels during menopause and perimenopause leads to a reduction in vaginal lactobacilli, an increase in pH, and a subsequent proliferation of microorganisms such as *E. coli*, thereby augmenting susceptibility to UTIs [65]. A retrospective analysis of older postmenopausal women found that a reduction in vaginal pH after short-term treatment with local estrogen decreased the incidence of rUTI [66]. The weakened immunity of older women also makes them more vulnerable to bacteria.

Genitourinary conditions related to ovarian aging and menopause often co-occur. For example, pelvic organ prolapse (POP) is frequently accompanied by UTIs, urinary incontinence, and vaginitis. To address the constellation of symptoms arising from estrogen deficiency, the term Genitourinary Syndrome of Menopause (GSM) was introduced in 2014 by the International Society for the Study of Women’s Sexual Health and the North American Menopause Society (NAMS). GSM encompasses symptoms such as dyspareunia, vaginal dryness, burning, itching, urinary urgency, recurrent UTIs, and painful intercourse [67]. Further research is essential to elucidate the interrelationships between UTIs, GSM, and ovarian aging and to explore interventions that could mitigate UTI and GSM by delaying the progression of ovarian aging.

#### 2.2.3 Chronic kidney disease (CKD)

The decline of renal function associated with aging is related to kidney fibrosis. In ovariectomized (OVX) mice, increased renal fibrosis was observed, an effect partially mitigated by estrogen therapy [68]. Estrogen supplementation reduced superoxide production and nitrotyrosine expression in the kidneys by downregulating AT1-receptor expression and upregulating AT2-receptor expression [68, 69]. A 10-year prospective study demonstrated that estrogen improved blood pressure and the urinary albumin-to-creatinine ratio in postmenopausal women without affecting glomerular filtration rates [70]. Additionally, estrogen and progesterone regulate renal ion reabsorption by modulating ion channels and pumps. OVX reduced the expression of cyclic nucleotide-gated A1 subunits (CNG-A1), a sodium channel in the renal cortex, and diminished both the expression and activity of Na^+^/K^+^ ATPase. Estrogen treatment reversed these changes, while progesterone restored Na^+^/K^+^ ATPase activity to baseline levels [71]. Estradiol also inhibited aquaporin-2 (AQP2), suggesting that fluctuations in female estrogen may disrupt water homeostasis [72].

CKD, characterized by renal fibrosis and nephron loss, often arises from age-associated conditions such as hypertension and diabetes. CKD progresses more slowly in women, with premenopausal diabetic women having a lower risk of end-stage kidney disease compared to age-matched diabetic men. However, this protective effect diminishes after menopause, coinciding with decreased estrogen levels and a relative increase in androgens, which exacerbate kidney disease progression [73, 74]. Moreover, women undergoing OVX before menopause are more susceptible to CKD [12]. Estradiol supplementation therapy has been shown to reduce extracellular matrix precipitation in glomerular and mesangial cells of diabetic animals, protect podocytes from damage, ameliorate age-related glomerular hypertrophy, increase nitric oxide (NO) production, and regulate vascular endothelial growth factor (VEGF) expression in tubular and vascular smooth muscle cells. These mechanisms collectively mitigate diabetic nephropathy and glomerular sclerosis [75-77]. Further research is needed to evaluate the potential of estrogen therapy, either alone or in combination with other medications, for clinical management of CKD.

### 2.3 Digestive system

#### 2.3.1 Age-related periodontal disease

Periodontal disease, which affects the supporting structures of the teeth, shows a strong association with aging, with its incidence increasing significantly with age. Evidence suggests that estrogen supplementation reduces periodontal inflammation, gum bleeding, and tooth loss in postmenopausal women [78]. This effect may be attributed to the modulation of inflammatory factors such as IL-6 and IL-1β, which are elevated in periodontal disease. Estrogen has been shown to suppress the inflammatory responses mediated by these factors [28, 79]. However, the relationship between periodontal disease and ovarian aging, as well as the precise mechanisms by which estrogen influences periodontal tissues, remains insufficiently explored. It is clear, nonetheless, that many age-associated diseases involve systemic inflammatory responses, which are exacerbated by estrogen deficiency.

#### 2.3.2 Hepatic aging

In the liver, hepatocyte senescence promotes fat accumulation and steatosis [80]. Non-alcoholic fatty liver disease (NAFLD), characterized by excessive hepatic fat deposition, can progress to non-alcoholic steatohepatitis (NASH), cirrhosis, and hepatocellular carcinoma [81]. Age and gender are critical factors in NAFLD, with estrogen playing a protective role [82]. Postmenopausal women show a prevalence of NAFLD twice that of premenopausal women [83]. This protective effect is supported by findings from human hormone replacement therapy and animal studies, demonstrating that estrogen can inhibit hepatic de novo lipogenesis and increase fatty acid oxidation [84]. Furthermore, ER-α levels inversely correlate with disease severity in NASH patients [85]. The relative increase in androgen levels during menopause contributes to a pro-atherosclerotic metabolic profile, potentially explaining the higher incidence of NAFLD in postmenopausal women [86]. In oophorectomized older rats, elevated oxidative stress and inflammation in the liver underscore the exacerbation of liver damage due to diminished ovarian function [87]. Additionally, the association between anti-Müllerian hormone (AMH) and NASH highlights the association between reproductive aging and the progression of NAFLD [13].

#### 2.3.3 Intestinal aging

The intestinal tract, a critical organ for nutrient absorption and immune function, is profoundly affected by aging, which disrupts intestinal microbiota balance and influences health and disease [88]. The intestinal microbiota interacts with sex hormones, and reductions in estrogen and progesterone—such as those observed after oophorectomy—can alter the gut microbiome, aggravate metabolic dysfunction, and compromise intestinal barrier integrity [89]. Estrogen and progesterone have also been shown to reduce the production of pro-inflammatory cytokines in epithelial barrier cells, thereby preventing inflammation-induced intestinal damage [90].

#### 2.3.4 Others

Pancreatic steatosis, another condition associated with aging, has been related to both age and menopause. A cross-sectional study revealed that women begin to experience significant increases in pancreatic fat infiltration at around 40 years of age [91]. Unfortunately, research investigating the relationship between ovarian aging and pancreatic aging remains scarce, warranting further exploration.

### 2.4 Cardiovascular system

#### 2.4.1 Age-related cardiac diseases

Aging is accompanied by progressive cardiac remodeling, which fosters the development of fibrosis and ventricular stiffness, thereby predisposing individuals to heart failure, arrhythmias, and sudden cardiac death [92]. Estrogen regulates the fibrotic response and the differentiation of myofibroblasts through ERs [93]. Specifically, ERβ inhibits angiotensin-II-induced cardiac fibrosis [94], while ERα protects against right ventricular diastolic dysfunction and fibrosis [95]. Estrogen replacement therapy can mitigate increased left ventricular remodeling in aging rats after OVX, which may be achieved by the regulation of angiotensin II receptor expression [96]. The decline in estrogen levels in postmenopausal women is linked to increased oxidative stress, contributing to left ventricular hypertrophy, which subsequently leads to diastolic dysfunction and heart failure with preserved ejection fraction [97]. By partially acting on the G protein-coupled estrogen receptor, estrogen inhibits cardiac mitochondrial reactive oxygen species (ROS) [97]. Sarcolemmal ATP-sensitive K^+^(K_ATP_) channels, essential for linking metabolic states to cardiomyocyte membrane excitability, are abundantly expressed in cardiomyocytes. Aging reduces cardiac sarcolemmal K_ATP_ channels exclusively in female hearts, whereas male hearts remain unaffected [98]. This reduction may be driven by an age-dependent decline in estrogen levels, which regulate the abundance of these channels [98]. Consequently, the decrease in channel number may explain the reduced stress tolerance observed in the cardiac muscle of older women.

#### 2.4.2 Vascular aging

Estrogen exerts broad protective effects on the vascular system. It regulates injury-induced chemokines, cytokines, and leukocyte infiltration while modulating growth factor expression and oxidative stress responses in damaged vessels [99]. For example, 17β-estradiol reduces the expression of cytokine-induced neutrophil chemokine-2β and suppresses C-reactive protein levels in injured arteries [100]. Furthermore, estrogen may elevate vascular function by increasing nitric oxide bioavailability and suppressing ROS levels [101]. However, estrogen deprivation following menopause markedly diminishes this protection, leading to a dramatic rise in cardiovascular disease (CVD) incidence in postmenopausal women compared to their premenopausal counterparts [102]. This transition is accompanied by a gradual increase in large elastic artery stiffness during early perimenopause [102]. The relationship between gonadal steroids, their protective effects, and ovarian aging warrants further investigation.

A study involving 3,108 women aged 20-60 years identified AMH as an independent predictor of CVD. A reduction of 1 ng/mL in log AMH levels was associated with a 21% increased risk of CVD and a 26% increased risk of coronary heart disease [103]. These findings underscore the importance of cardiovascular health monitoring in individuals with low AMH levels.

In summary, hormonal changes, particularly the decline in estrogen levels driven by ovarian aging, exacerbate vascular aging and structural changes in cardiac tissue among elderly women. These changes contribute to cardiovascular dysfunction, increasing the risk of conditions such as hypertension, coronary heart disease, and heart failure.

### 2.5 Nervous system

Estrogen exerts multifaceted neuroprotective effects, including the regulation of synaptogenesis, enhancement of cerebral blood flow, mediation of neurotransmitter activity, inhibition of apoptosis, and provision of anti-inflammatory and antioxidant benefits [104]. It also regulates specific cognitive functions, such as working memory and language acquisition [104]. Estradiol achieves these effects partly by activating the PI3K signaling pathway [105]. Oxidative stress, a significant contributor to age-related nerve damage, is amplified through androgen-induced activation of the membrane androgen receptor (mAR)-NADPH oxidase (NOX) signaling pathway, which is closely related to several neurodegenerative diseases [106]. The marked decline in estrogen levels during perimenopause and postmenopause, coupled with a relative rise in androgen levels, intensifies the activation of this pathway, resulting in more pronounced neural damage in women compared to age-matched men.

#### 2.5.1 Alzheimer’s Disease

Alzheimer’s disease (AD), an age-associated neurodegenerative disorder characterized by amyloid β deposition and tau pathology, leads to progressive dementia and severely diminished quality of life [107, 108]. A brain imaging study found that perimenopausal and postmenopausal women showed a decreased brain metabolism and mitochondrial cytochrome c oxidase activity in the same brain regions compared to premenopausal women, with the most pronounced changes occurring after menopause [109]. Estrogen deprivation has been shown to impair mitochondrial function in the brain, exacerbating oxidative stress and β-amyloid and Aβ-binding-alcohol-dehydrogenasev accumulation, as evidenced by OVX mouse models [110]. Estradiol supplementation reverses these mitochondrial dysfunctions, restoring brain bioenergetics and mitigating oxidative damage [110]. A clinical sex dimorphism has been identified in the predisposition to AD, with women undergoing bilateral oophorectomy before menopause showing nearly double the risk of dementia, suggesting that the precipitous drop in endogenous estrogen levels during menopause may act as a trigger for AD [111, 112]. Another major pathological feature of AD is the formation of neuronal tangles caused by excessive phosphorylation of Tau protein. The phosphorylation of Tau protein in the hippocampus of OVX rats gradually increased, accompanied by changes in associative learning [113]. OVX altered imaging biomarkers associated with AD in mice, including increased hypothalamic osmotic pressure regulation and energy metabolism biomarkers, decreased white matter integrity, and reduced resting-state functional connectivity [114].

Progesterone also influences neuroinflammation, primarily by inhibiting NLRP3-Caspase-1 activation, upregulating autophagy, and suppressing IL-1β secretion in astrocytes [115]. Its neuroprotective actions are mediated through MAPK and Akt signaling pathways [116]. However, contrasting effects of synthetic progestins such as medroxyprogesterone acetate have been observed, highlighting the critical need for careful selection of hormone replacement therapies to optimize neuroprotection [117].

Emerging evidence implicates FSH in AD pathogenesis. Cerebral neurons expressing FSH receptors have been shown to exacerbate amyloid β deposition and tau pathology via the C/EBPβ/δ-secretase pathway, with cognitive decline mitigated by FSH blockade [118]. Similarly, luteinizing hormone (LH) inhibition improves cognitive outcomes and reduces neuronal damage in AD models [119].

#### 2.5.2 Parkinson's Disease

Parkinson’s disease (PD), another prominent neurodegenerative disorder, is characterized by the progressive degeneration of dopaminergic neurons in the substantia nigra, manifesting as tremors and rigidity [120]. Estrogen provides neuroprotection in PD through MAPK and Akt pathways, as well as by regulating monoamine oxidase activity and reducing IL-6-mediated neuroinflammation [121, 122]. Its promotion of autophagy maturation in the dopaminergic regions of the brain through the ERK pathway has been associated with protective effects in rotenone-induced PD models [123]. Estrogen may also suppress the nigral renin-angiotensin system and NOX activity, preserving dopaminergic neurons and explaining the lower PD risk in premenopausal women [124].

Similar to AD, clinical trials of hormone supplementation therapy associated with PD have been mixed. For example, one study found that the combination of esterified estrogen and progesterone increased the risk of PD, while conjugated estrogen with progesterone had no association with the risk of PD [125]. The specific reasons remain to be explored. Some studies suggested that estrogen had no protective effect on PD or had potential benefits on the motor and cognitive functions of PD patients [126-128]. A case-control study found that postmenopausal hormone replacement therapy was associated with a lower risk of PD [129].

The timing of estrogen supplementation significantly influences its neuroprotective efficacy. Delayed administration after prolonged estrogen deficiency can negate its beneficial effects, whereas early initiation post-OVX attenuates ischemic stroke-induced inflammation and improves outcomes [130, 131]. This underscores the importance of identifying the therapeutic window for hormone replacement therapy, as early postmenopausal intervention may yield positive outcomes, while late initiation could prove detrimental.

#### 2.5.3 Age-related macular degeneration (AMD)

Beyond neurodegenerative diseases, estrogen deficiency has been implicated in age-related macular degeneration (AMD), a chronic retinal condition leading to blindness in the elderly [132]. Older women are disproportionately affected, with a risk of developing dry AMD that is twice as high as men and a greater likelihood of severe disease progression, largely attributed to the decline in estrogen levels [133]. The pathophysiology of AMD involves persistent oxidative stress, chronic inflammation of the retinal pigment epithelium (RPE), lipofuscin accumulation within RPE cells, and the formation of drusen deposits between the RPE and Bruch’s membrane [134]. Estrogen has been shown to modulate RPE function, enhancing cell survival under oxidative stress by suppressing the upregulation of H_2_O_2_-induced apoptotic proteins and regulating matrix metalloproteinase-2 (MMP-2) activity [133, 135]. As a key enzyme in the degradation of extracellular matrix components such as laminin and collagen types I and IV, MMP-2 is essential for the structural integrity of Bruch’s membrane. These findings suggest that postmenopausal estrogen deficiency may increase susceptibility to AMD, with chronic inflammation serving as an additional contributing factor. Genetic studies further support the link between ovarian aging and AMD. Jiang *et al.* identified a cluster of five single nucleotide polymorphisms (SNPs) on the X chromosome associated with AMD, overlapping with the DIAPH2 gene, which is implicated in premature ovarian failure (POF) [136]. This genetic association hints at a potential connection between POF and AMD, though clinical validation is lacking. Moreover, epidemiological studies suggest that hormone replacement therapy may offer a protective effect against AMD, particularly its exudative subtype [137].

#### 2.5.4 Age-related hearing loss (ARHL)

Hearing loss is another prevalent condition in the elderly, with growing evidence of its association with estrogen deficiency. By binding to ERα and ERβ, estrogen activates the MAPK pathway, promoting neuronal survival through anti-apoptotic mechanisms and influencing mitochondrial activity via calcium signaling. These pathways are vital for preserving the auditory neurophysiological adaptations required for sound processing [138]. Estrogen also modifies IGF-1R and promotes vascular endothelial growth factor (VEGF) secretion through the PI3K/AKT pathway, thereby supporting the health of sensory cells in the cochlea. Estrogen supplementation has been associated with a reduction in the auditory brainstem response threshold, reflecting its protective role in auditory function [139, 140]. Interestingly, hearing loss has been identified as a risk factor for dementia [141], suggesting it may serve as an early indicator of estrogen deficiency or menopause-related neurodegeneration. However, the impact of progesterone on ARHL remains controversial, warranting further investigation.

### 2.6 Respiratory system

With advancing age, senescent cells progressively accumulate in various lung cell types, including epithelial, endothelial, fibroblast, and immune cells [142]. This senescent phenotype is marked by reduced bronchial density, increased bronchial diameter, diminished alveolar surface area, and enlarged alveoli and airspaces, coupled with a gradual loss of lung elasticity—even in the absence of overt disease [142]. Consequently, the lungs become more vulnerable to chronic inflammation and pulmonary fibrosis, which are associated with age-related diseases such as chronic obstructive pulmonary disease (COPD), non-cystic fibrosis bronchiectasis, and idiopathic pulmonary fibrosis (IPF).

### 2.6.1 Chronic obstructive pulmonary disease (COPD)

COPD, a persistent inflammatory lung disease, encompasses conditions such as emphysema and chronic bronchitis [143]. These are characterized by irreversible or partially reversible airway obstruction, reduced lung elasticity, and alveolar enlargement [144]. Ovarian aging may directly or indirectly affect lung structure and function. Ovariectomy studies in animal models have demonstrated that the loss of ovaries and reproductive hormones detrimentally impacts alveolar morphology, resulting in a reduced alveolar count [145]. On the contrary, progesterone mitigated airway remodeling and glucocorticoid resistance in mice exposed to ozone [146]. Oxidative stress, a central mechanism in COPD pathogenesis [147], is alleviated by progesterone through its ability to lower cellular infiltration in bronchoalveolar lavage fluid, increase antioxidant enzyme activity, and reduce lipid peroxidation marker MDA levels in lung tissue [148]. These findings indicate that progesterone alleviates inflammation and oxidative stress associated with COPD, while facilitating the repair of lung tissue damage. Additionally, progesterone also exerts protective effects by regulating the c-MYC/SIRT1/PGC-1α pathway [148]. Estrogen replacement therapy has been observed to promote alveolar regeneration in ovariectomized mice, suggesting its potential to ameliorate COPD [149]. Hence, decreased progesterone and estrogen levels due to ovarian aging may diminish this protective effect. Postmenopausal women exhibit significantly lower forced expiratory volume in 1 second (FEV_1_) and forced vital capacity (FVC) than premenopausal women [150-153]. A longitudinal cohort study involving 2,020 participants over 21 years reported reduced FVC, FEV_1_, and forced expiratory flow 25%-75% (FEF_25-75_) in postmenopausal women compared to their premenopausal counterparts [151]. Similarly, the UK Biobank study of 141,076 women found lower FVC and FEV_1_ and increased spirometric restriction (FVC < lower limit of normal) in postmenopausal women, with these effects being more pronounced in women with hysterectomy or oophorectomy [152]. Earlier menopause further exacerbated the decline in lung function [152]. Another study from the European Community Respiratory Health Survey reported accelerated decreases in FVC and FEV_1_ among transitional and postmenopausal women compared to those with regular menstrual cycles [153].

The age of ovarian senescence also influences COPD risk. A large UK cohort study of 271,271 women found an elevated risk of COPD-related hospitalization or death among those experiencing early menopause (<47 years) [154]. Similarly, an Australian study of 11,258 women reported an association between premature menopause (≤40 years) and COPD risk [155]. Women experiencing menopause before 40 exhibited a hazard ratio (HR) of 1.69 (95% CI 1.63-1.75) for COPD, while those aged 40-44 had an HR of 1.42 (95% CI 1.38-1.47) [15]. Bilateral oophorectomy before 46 years was also associated with heightened COPD risk, even among non-smokers [156, 157].

### 2.6.2 Bronchiectasis and idiopathic pulmonary fibrosis

Both bronchiectasis and IPF exhibit gender disparities, suggesting hormonal influence. A later onset of menopause is associated with a reduced risk of bronchiectasis (HR 0.90, 95% CI 0.84-0.96 for ≥55 years vs. <40 years) [158]. Non-cystic fibrosis bronchiectasis, a complex chronic respiratory disease characterized by excessive sputum production and permanent abnormal bronchial dilatation, has the highest prevalence in postmenopausal women [159]. Epidemiological studies indicate that gender is associated with idiopathic pulmonary fibrosis (IPF), with men experiencing greater negative impacts and women exhibiting higher survival rates, which implies a protective role of estrogen [160, 161]. Estrogen may ameliorate pulmonary fibrosis by modulating TGF-β1-induced signaling mechanisms [162]. Dietary phytoestrogens may impede the onset of pulmonary fibrosis [163].

### 2.7 Endocrine system

The accumulation of senescent cells in adipose tissue and senescence of pancreatic β-cells have been reported to increase chronic adipose tissue inflammation and insulin resistance, leading to obesity and type 2 diabetes mellitus (T2DM) [164]. Moreover, menopause, as an aging process in women, is associated with an increased risk of metabolic disorders including obesity and T2DM [165]. The reduction in estrogen levels leads to a variety of changes in sugar and fat metabolism [166].

### 2.7.1 Obesity

Based on recent data from the World Health Organization, approximately 13% of adults globally are classified as obese [167], with projections indicating that by 2030, 50% of adults in the United States will fall into the obese category [168]. Females exhibit a higher susceptibility to obesity compared to males, particularly within the middle-aged and older demographic cohorts [169, 170]. This disparity could potentially be attributable to ovarian aging, as menopause accelerates the accumulation of central obesity when compared to the premenopausal period [10]. The physiological and metabolic changes associated with menopause are primarily driven by estrogen deficiency, which has been shown to directly influence lipid metabolism [171]. Reductions in estrogen levels may specifically result in the accumulation of central fat and the promotion of visceral adiposity [6]. Animal models and studies using gonadotropin-releasing hormone agonists to suppress ovarian function in humans have demonstrated an elevation in visceral fat mass, without a corresponding change in total fat mass. The reintroduction of estrogen reverses the increase in visceral fat, further supporting the role of estrogen in fat distribution [172, 173]. Additionally, the rise in FSH levels due to the decline in estradiol has been associated with weight gain, particularly in the visceral fat compartment. FSH may facilitate fat redistribution and promote a pro-inflammatory environment that exacerbates adiposity [6, 172, 174]. Estrogen deficiency also impacts energy balance, with studies showing that estrogen suppresses appetite and regulates energy intake [175]. In OVX animals, a reduction in energy expenditure is observed, even when energy intake remains unchanged [176, 177]. Furthermore, early menopause has been associated with an increased risk of obesity, suggesting that the timing of menopause influences long-term metabolic health [178]. Nevertheless, the effects and mechanisms of the ovarian aging process on obesity need to be further investigated.

### 2.7.2 Type 2 diabetes mellitus

Both aging and obesity significantly contribute to the development of T2DM [179, 180]. As discussed previously, menopause influences adipose tissue distribution and accumulation, leading to increased cytokine production and peripheral insulin resistance, which contribute to low-grade systemic inflammation [181]. Apart from obesity, ovarian aging further elevates the occurrence of dysglycemia [182]. The relative androgenic state caused by a substantial decrease in ovarian estrogen and little effect on androgen production during menopause is accompanied by unfavorable metabolic features that may predispose to T2DM [14, 183]. Menopausal estrogen decline leads to susceptibility to T2DM by affecting insulin behavior and insulin secretion as well as degradation. Changes in insulin behavior involve increased visceral adiposity, elevated formation of free fatty acids with their transformation into triglycerides in muscle and liver, chronic inflammation, modified levels of sex hormones, and impaired insulin signaling due to diminished activation of ERα. These alterations contribute to insulin resistance and increase the predisposition to T2DM [184]. Alterations in insulin secretion and degradation include the apoptosis of pancreatic beta cells, reduced insulin secretion, and heightened hepatic insulin degradation. With time, the pancreas becomes incapable of satisfying the escalated insulin requirements, culminating in the onset of T2DM [184].

Epidemiological studies provide strong evidence linking early menopause and POI to an elevated risk of T2DM later in life. A cohort study of 36,931 women from Taiwan found that those with POI or early menopause (defined as menopause before 45 years) had a significantly higher risk of diabetes-related mortality compared to those who experienced menopause between ages 50-55 (HR 1.44, 95% CI 1.03-2.02) [185]. A prospective study conducted over 11 years showed that women with POI had a 32% higher risk of developing T2DM than those without [186]. Additionally, a meta-analysis of 13 studies involving 191,762 women found that both early menopause and POI were associated with a significantly higher risk of T2DM compared to women who entered menopause after 45 years (OR: 1.12, 95% CI 1.01-1.20 for early menopause; OR: 1.53, 95% CI 1.03-2.27 for POI) [187].

### 2.8 Integumentary system

Relative to other menopausal symptoms, skin aging, and alopecia have received less attention. Skin and hair follicles are one of the main targets of sex hormone action [188, 189]. ERs are plentiful in the human dermis and epidermis, including the scalp [190, 191]. Multiple changes in skin action due to a substantial decline in estrogen during menopause [192-194]. The impaired skin barrier function results in decreased skin moisture and sebum production on the face and scalp, leading to dryness. Additionally, a decrease in antioxidant activity hinders wound healing, while diminished collagen and elastin synthesis, along with lower levels of glycosaminoglycans in the extracellular matrix, further contribute to skin wrinkling and dermal thinning. Estradiol, a key regulator of the hair cycle, extends the anagen phase and promotes hair growth by stimulating the production of growth factors essential for the proliferation of keratin-forming cells in hair follicles [195]. Consequently, reduced estradiol levels during menopause are associated with decreased hair renewal, growth, and thickness, leading to hair thinning.

Compelling evidence was also observed in cohort studies. Skin collagen levels have been shown to decline significantly, with a 30% reduction observed within the first five years of menopause, followed by an additional annual decline of 2% over the subsequent 15 years [196]. Over 64% of women reported skin problems in menopause with skin dryness being the predominant complaint and half of them believed that menopause was the main cause [197]. In addition to skin dryness, loss of skin elasticity (70.1% vs. 31.4%), pigmentation spots (57.9% vs. 26.4%), and wrinkles (60.8% vs. 30.8%) occur more frequently in menopausal women than in those who are still menstruating (All *P* < 0.001) [11]. In the perimenopausal period, women predisposed to centripetal alopecia may undergo notable hair thinning. Menopausal hair-related issues encompass a reduction in scalp hair density coupled with an augmentation in facial hair growth (unwanted) [198]. Overall, menopause is an easily overlooked contributing factor to skin aging and hair loss.

### 2.9 Immune system

The immune system is intricately related to the aging process, undergoing significant remodeling throughout the lifespan [199]. Age-related immune deficiency and immune system reconfiguration are evident in older individuals. Among menopausal women, the estrogen deficiency characteristic of this stage contributes to an increased prevalence of infections and autoimmune disorders, as estrogen acts as a stimulator of humoral immunity. In postmenopausal women, there is a notable observation of elevated levels of pro-inflammatory serum markers such as IL-1, IL-6, and TNFα. This inflammatory profile is accompanied by heightened cellular responses to these cytokines, reduced responses to pathogens or stimuli, lower counts of CD4+ T lymphocytes and B lymphocytes, and diminished cytotoxic activity of natural killer (NK) cells, compared to premenopausal women [200, 201]. The diminished immune response contributes to increased susceptibility to new human papillomavirus (HPV) and human immunodeficiency virus (HIV) infections [202, 203]. Furthermore, postmenopausal women also exhibit an increased susceptibility to autoimmune diseases or an exacerbation of existing autoimmune disease symptoms [204]. The causal relationship between ovarian aging and immune senescence has yet to be definitively established, necessitating further exploration and clarification of the underlying mechanisms.

## 2.10 Reproductive system

### 2.10.1 Vaginal and vulvar aging

The vagina and vulva are key target organs for estrogen. After menopause, a remarkable reduction in estrogen levels results in vulvovaginal atrophy and atrophic vaginitis, emerging as the predominant manifestations of vaginal and vulvar aging. The molecular process underlying this aging involves a reciprocal relationship influenced by both estrogen and microbiota. The post-menopausal decline in estrogen leads to the depletion of vaginal epithelial cells and atrophy of the genital tract [205]. These changes manifest as a shortened and narrowed vagina, along with a thin and dry vaginal epithelium, as well as hypersensitivity or decreased sensation in the vagina, which are the primary symptoms of vaginal aging [206]. Estrogen causes the production of glycogen by vaginal epithelial cells, whereas its decline during menopause reduces glycogen synthesis, disrupting the vaginal microbiome by decreasing lactobacilli populations and raising vaginal pH [207]. This cascade of pathophysiological changes contributes to broader alterations in the genitourinary system. Key features include pallor and fragility of the vaginal mucosa, reduced vaginal secretions, shifts in microbial community composition, diminished pubic hair, loss of subcutaneous fat in the labia majora, and a reduction in the volume of the labia minora and vestibular glands [208]. Collectively, these factors underpin the processes driving vaginal and vulvar aging.

### 2.10.2 Pelvic organ prolapse (POP)

POP is defined as the vaginal wall and/or uterus drops from their normal anatomical position into or beyond the hymen remnant, typically caused by excessive relaxation of the pelvic connective tissue and supporting ligaments [209]. Age is a critical factor in POP, with the prevalence of symptomatic POP in China reported to be eight times higher in women aged over 70 years compared to those aged 20-29 years [210]. POP is associated with decreased total collagen content and collagen solubility [211]. Premenopausal women with POP had significantly lower serum estrogen levels and ER levels in the uterine ligaments [212]. Experimental studies provide additional insights into the role of estrogen in POP. Estradiol therapy in rhesus monkeys has been shown to upregulate mRNA expression of collagen types I and III and stimulate collagen synthesis in pelvic floor connective tissue [213]. In addition, changes in pelvic floor ER expression may alter the risk of pelvic organ prolapse. Postmenopausal women exhibit higher ERα expression, and elevated ERα/ERβ ratios have been observed in both premenopausal and postmenopausal women with POP [214]. However, the influence of hormones secreted by the ovaries on POP is an area that requires further investigation. This could potentially yield innovative concepts for the prevention and treatment of POP.

## 3. Treatment strategies for both ovarian aging and age-related diseases

**Figure 3.**
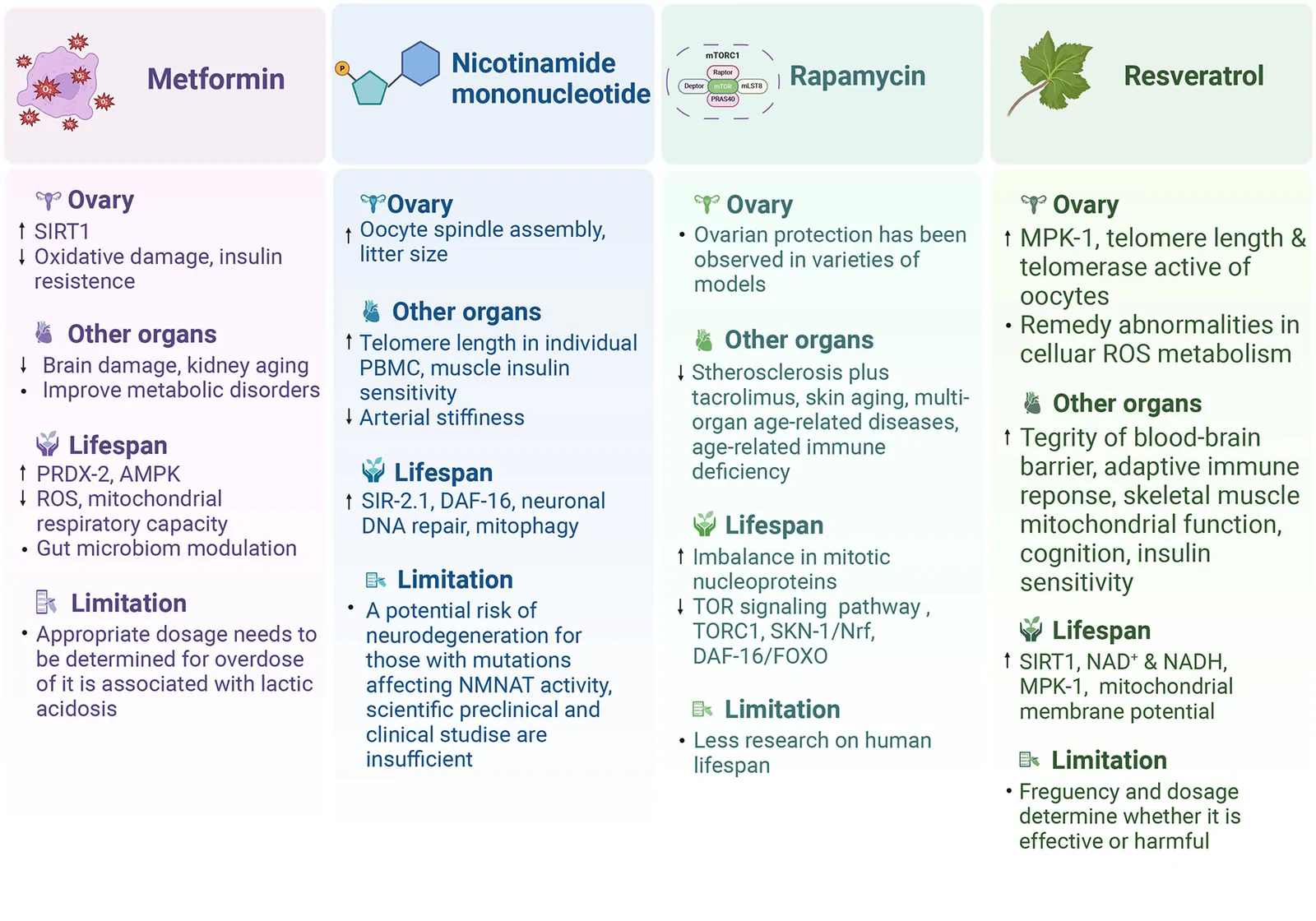
**Treatment strategies for both ovarian aging and age-related diseases**. Created with BioRender.com.

The aging process, whether ovarian or organic, is inevitable. However, it is feasible to attenuate aging by extending lifespan and fostering healthy aging. The concept of “healthy aging” represents a novel concept that characterizes aging as the simultaneous maintenance of health across various physiological systems [215, 216]. This notion excludes life-altering diseases and prioritizes a high quality of life as an individual’s age, ensuring freedom from severe ailments and disabilities while maintaining robust physical and cognitive capabilities. This perspective has sparked a new era in aging research. Ameliorating reproductive aging and age-related diseases can promote healthy longevity [3]. Since the 1930s, when biologists initially discovered that restricting caloric intake could extend the lifespan of mice and rats, researchers have delved into various pharmacological interventions aimed at decelerating the aging process [217]. Currently, pharmacologic strategies are considered the principal approach for delaying aging. In this article, we discuss the anti-aging medications that have the potential to slow both ovarian and somatic aging processes (Fig. 3).

### 3.1 Metformin

As a first-line oral biguanide antidiabetic agent, metformin lowers blood glucose levels by enhancing insulin sensitivity, reducing glucose absorption in the small intestine, and decreasing hepatic glycogen synthesis. Following its widespread adoption as a first-line treatment for diabetes, metformin was identified as a potential modulator of both health span and lifespan in various animal models [218, 219]. The lifespan-extending effects of metformin are mediated through the activation of AMP-activated protein kinase (AMPK) via inhibition of v-ATPase, independent of alterations in cellular AMP level [220]. This is particularly significant as AMPK activity is often diminished during aging [221]. Moreover, metformin depresses mitochondrial respiratory capacity, thereby downregulating TOR complex 1 (TORC1) kinase activity. This suppression increases the transcription of acyl-CoA dehydrogenase family member 10 (ACAD10), which inhibits growth and extends lifespan [222, 223]. Enhanced PRDX-2 expression in mitohormesis and thus potential activation of the longevity signaling pathway, also contribute to the lifespan-prolonging effects of metformin [224]. Additionally, metformin can attenuate ROS levels, thereby reducing oxidative damage and senescence-associated inflammatory factors [225, 226]. In both *C. elegans* and mice, metformin increased their lifespan and health span [218, 219]. Beyond its systemic benefits, metformin effectively prevents brain damage caused by cerebral ischemia and delays stroke onset in spontaneously hypertensive rats with stroke [227]. In addition, metformin delays kidney aging, reduces cardiovascular risk events, and meliorates T2DM [228-230]. In humans, metformin may enhance longevity by modulating the gut microbiome [231]. Clinical trials have further substantiated the metformin’s potential life-prolonging properties. For example, a randomized, double-blind, placebo-controlled trial demonstrated that six weeks of metformin administration improved age-related metabolic derangements in older adults with glucose intolerance [232]. Furthermore, another randomized clinical trial indicated that metformin improved amplified effector pathways in patients with prediabetes, which had been demonstrated to modulate longevity in animal models [233]. Metformin’s role in enhancing quality of life in aging populations includes its ability to improve organ function. Specifically, it supports bone health by protecting joints and reducing the risk of fracture [234]. Additionally, metformin contributes to improved coronary function by decelerating plaque accumulation and enhancing cardiac performance through the reduction of oxygen demand and left ventricular mass [234].

For ovarian aging, metformin has also demonstrated anti-aging properties. In a study involving 27-week-old female C57BL/6 mice, a six-month course of metformin treatment significantly elevated serum estradiol levels and increased the proportion of regular estrous cycles in the treated group compared to controls [235]. Metformin-treated mice also exhibited a greater number of primordial and primary follicles, along with enhanced expression of SIRT1, suggesting a mechanism involving SIRT1 upregulation and reduced oxidative damage. Furthermore, findings from a randomized trial revealed that low-dose metformin enhanced pregnancy rates in individuals undergoing in vitro fertilization (IVF) cycles who did not have polycystic ovary syndrome, probably by mitigating insulin resistance [236]. Despite its potential, the side effects of metformin must be carefully considered. High doses of metformin can lead to lactic acidosis, characterized by elevated lactate levels, and its use is contraindicated in individuals with heart failure, chronic kidney disease, liver dysfunction, or other conditions that increase the risk of lactic acidosis [237]. Further studies are essential to investigate the anti-ovarian aging effects of metformin in humans and to determine the optimal dosage regimen.

### 3.2 Nicotinamide mononucleotide

Nicotinamide mononucleotide (NMN) is a biologically active nucleotide and a crucial precursor for nicotinamide adenine dinucleotide (NAD)^+^ biosynthesis, located in the nucleus, cytoplasm, and mitochondria [238]. As a core coenzyme of energy metabolism and an indispensable redox cofactor, NAD^+^ homeostasis is essential for the maintenance of normal physiological functions [239]. By activating key proteins such as SIR-2.1 and DAF-16, NAD affects lifespan [240]. However, NAD^+^ levels inevitably decline with age, and pharmacological supplementation to restore NAD^+^ levels has been shown to rescue age-related metabolic decline and prolong the lifespan of nematodes [240]. Moreover, supplementation of NAD^+^ precursors has been revealed to promote mitochondrial homeostasis [241]. Specially, the restoration of intracellular NAD^+^ levels by NMN supplementation attenuates neuropathology associated with ataxia telangiectasia, improves neuromuscular function, delays memory decline, and prolongs the lifespan in *atm-1* mutant worms by facilitating neuronal DNA repair and improving mitochondrial quality via mitophagy [242]. Reduced NAD^+^ levels are implicated in the predisposition to age-related diseases, including metabolic dysfunction, inflammation, and neurodegenerative disorders, conditions that may be alleviated through NMN supplementation [243]. Three trials demonstrated the safety and efficacy of oral NMN, with healthy individuals tolerating up to 1250 mg of NMN per day for 4 weeks [244-246]. Especially, the daily oral administration of 600 mg of NMN in healthy adults resulted in significant improvements in general health and physical status, as evidenced by reductions in biological age markers in the blood, enhancements in 36-Item Short Form Survey (SF-36) scores, and increased scores on the six-minute walking test [247]. Additionally, NMN demonstrated the potential in alleviating arterial stiffness [248]. NMN improved muscle insulin sensitivity, remodeling, and insulin signaling in overweight or obese pre-diabetic women [249] and significantly prolonged telomere length in individual peripheral blood mononuclear cells (PBMC) [250]. Notably, the timing of NMN administration may also have an impact on its efficacy. The administration of NMN in the afternoon demonstrates greater efficacy in improving lower limb function and reducing drowsiness in older adults [251]. NMN supplementation has also demonstrated a protective effect against ovarian aging in a mouse model. NMN improves oocyte quality by improving spindle assembly and increasing litter size [252, 253]. While NAD+ precursors like NMN are generally considered safe for most individuals, caution is warranted for those with mutations affecting nicotinamide mononucleotide adenylyl transferase (NMNAT) activity, as they may be at increased risk for neurodegeneration when consuming such supplements [254]. Moreover, the safety and efficacy of the various NMN supplements available on the market remain debatable due to insufficient scientific preclinical and clinical studies.

### 3.3 Rapamycin

Rapamycin (sirolimus), a natural antifungal macrolide, was originally isolated from soil samples on Easter Island in the late 1960s and is synthesized by the bacterium *Streptomyces hygroscropicus* [255]. In addition to its well-established role as an anti-inflammatory agent, rapamycin is clinically approved for preventing tissue rejection following organ transplantation and as an adjunct in cancer therapy [256]. In 2009, rapamycin was first found to prolong the maximum lifespan of female mice by 14% and male mice by 9% [257]. Additionally, treatment with rapamycin significantly increased the lifespan of *C. elegans* compared to controls [258]. This longevity-promoting effect is primarily mediated through the inhibition of the TOR signaling pathway, which is implicated in disease and aging process [259]. The genetic inhibition of TORC1, SKN-1/Nrf, and DAF-16/FOXO by rapamycin results in the activation of protective genes, thereby facilitating stress resistance and longevity [260]. In addition, the obstruction of TOR signaling by rapamycin modifies nuclear DNA translation, causing an imbalance in mitotic nucleoproteins and prolonging the average lifespan in a ubl-5-dependent manner [261]. Rapamycin ameliorates multiple organ aging-related diseases, including Parkinson’s disease, penal aging, myocardial fibrosis, carotid artery dysfunction, COPD, and diabetes [262]. Beyond these, the protective effect of rapamycin on aging ovaries was also observed in various rodent models, including mice and rats [263-266].

Though the longevity-enhancing effects of rapamycin have been documented across several species, including yeast, nematodes, fruit flies, and mice [267], there is still limited research investigating the impact of rapamycin on human aging. In the context of renal transplantation, the combination of rapamycin and tacrolimus has been found to more significantly reduce atherosclerosis compared to the combination of tacrolimus and mycophenolate [268]. A randomized controlled study indicated that rapamycin significantly improved clinical skin appearance and indicators of skin aging in individuals aged over 40 years [269]. Furthermore, rapamycin might ameliorate age-associated immune deficiency and reduce infections in the elderly. Notably, low-dose mTOR inhibitors have been shown to substantially reduce infection rates and augment the response to influenza vaccination, as well as the upregulation of antiviral gene expression in older participants vaccinated against seasonal influenza [270]. Presently, clinical trials are underway to explore the potential of rapamycin in reducing the incidence or elevation of aging-related biomarkers (NCT05237687). These studies also aim to evaluate the safety and efficacy of the rapalog everolimus in improving age-related physiological and molecular markers (NCT05835999) and to assess the long-term effectiveness of rapamycin in reducing clinical indicators of aging (NCT04488601). It is anticipated that future research will yield additional evidence regarding the impact of rapamycin on human longevity, somatic aging, and aging-related diseases.

### 3.4 Resveratrol

Resveratrol, a naturally occurring polyphenol found in red grapes, berries, wine, soybeans, and peanuts, possesses a range of biological activities, including antioxidant, hypotensive, hypolipidemic, immunomodulatory, and anti-inflammatory effects [271]. Additionally, resveratrol activates the longevity-associated protein Sirtuin 1 (SIRT1), resulting in a reduction of lipofuscin and ROS accumulation, thus extending lifespan [272]. Furthermore, resveratrol replenishes the levels of NAD^+^ and NADH and enhances mitochondrial membrane potential [273]. Resveratrol prolongs longevity in nematodes and mice, and ameliorates aging-related diseases in non-human primates [274]. A randomized, double-blind, placebo-controlled trial indicated that resveratrol is well tolerated at daily doses ranging from 300 to 1,000 mg in overweight individuals over 65 years of age, with no adverse effects on blood chemistry [275]. Moreover, a 26-week course of resveratrol supplementation improved memory and hippocampal functional connectivity in older adults over 50 years [276]. For older glucose-intolerant adults, resveratrol supplementation has a positive impact on vascular function [277]. Resveratrol has been shown to preserve blood-brain barrier integrity and stimulate adaptive immune responses, potentially enhancing the brain's resistance to amyloid deposition [278]. By harmonizing both peripheral and central immune responses, resveratrol may help decelerate the rapid cognitive decline associated with aging and menopause, particularly in elderly women. In another randomized, double-blind trial, regular low-dose resveratrol supplementation improved cerebrovascular function, cognition, and insulin sensitivity in middle-aged and older postmenopausal women [279]. Additionally, in older adults with functional limitations, the combination of exercise and resveratrol potentially increases skeletal muscle mitochondrial function and improves mobility and physical function [280]. Brown *et al*. discovered that resveratrol improved glucose metabolism and cardiovascular health [281]. Additionally, it offers neuroprotective and antidiabetic properties and might slow cognitive decline in AD patients [281]. Resveratrol supplementation was associated with a significant reduction in pain and improvements in overall health, including quality of life and cardiovascular function, when compared to placebo treatment [282].

Resveratrol has been found to extend the reproductive lifespan. In nematodes, resveratrol alleviates oxidative stress-induced damage and delays age-related reproductive decline by activating mitogen-activated protein kinase-1 (MPK-1), a homolog of the human ERK protein [273]. Long-term oral administration of resveratrol in mice has been reported to improve reproductive aging and fertility, as evidenced by increased oocyte quantity and quality, as well as enhanced telomere length and telomerase activity [283]. Moreover, resveratrol has been shown to ameliorate cellular ROS metabolism abnormalities, thereby improving oocyte quality in mice [284]. In a mouse model of POF, resveratrol treatment resulted in increased body and ovarian weight, enhanced follicle count, and reduced follicular atresia [285]. It was observed that low doses of resveratrol improved oocyte quality and ovarian function, whereas high doses resulted in embryonic apoptosis [283]. Thereby, although resveratrol demonstrates promising potential for addressing somatic and ovarian senescence, further studies are necessary to ascertain the optimal dosage and frequency of administration for human applications.

### 3.5 Other anti-aging drugs

The accumulation of reactive oxygen species (ROS) induces oxidative stress, which alters the cellular microenvironment, contributing to cellular senescence and a decline in both oocyte quality and quantity [286, 287]. Antioxidants, including vitamins A, C, and E, have been suggested to counteract oxidative damage and potentially delay aging. Notably, vitamin C has been associated with longevity in certain animal models. Research has demonstrated that middle-aged and elderly individuals tend to have lower plasma levels of vitamin C compared to younger populations [288]. The beneficial effects of vitamins on the female reproductive system encompass the maintenance of ovarian reserve function, improvements in the number of primordial and healthy follicles, reduction in atretic follicles, and enhancements in litter size and estrous cycle regularity [3]. However, there are associated side effects with vitamin use, particularly concerning female reproduction. Long-term administration of high-dose vitamins C and E can have detrimental effects on ovarian and uterine function [289]. Excessive intake of vitamins A and E may even raise the risk of mortality [290].

In addition to ROS, alterations in mitochondrial characteristics associated with aging, such as mitochondrial DNA mutations, changes in membrane potential, and a decline in metabolism, contribute to impaired mitochondrial function and are implicated in the aging process [286]. Given that oocytes are abundant in mitochondria, aging-related mitochondrial dysfunction exacerbates ovarian aging and infertility [291]. Coenzyme Q10 (CoQ10), a crucial component of the mitochondrial respiratory chain that declines with age, has been demonstrated to mitigate somatic and ovarian aging by enhancing mitochondrial function. The supplementation of external forms of CoQ10 to CoQ10-deficient human dermal fibroblasts has been observed to reverse the aging phenotype caused by CoQ10 deficiency [292]. Long-term supplementation with CoQ10 has the potential to increase overall longevity by reducing the risk of death from cardiovascular disease (including heart attack and stroke) and reducing all-cause mortality [293]. In murine models, CoQ10 treatment has been shown to enhance mitochondrial activity in aged mice by delaying the depletion of ovarian reserve, restoring oocyte mitochondrial gene expression, and improving overall mitochondrial function [294]. Increased expression of mitochondrial ROS scavenger was observed in aged mice treated with CoQ10 [294]. In humans, elevated CoQ10 levels correlate with improved embryo quality and higher pregnancy rates [295]. Oral supplementation of CoQ10 enhances the oxidative metabolism in follicular fluid and improves oocyte quality in women over 35 years of age [296]. A controlled randomized trial showed that oral coenzyme Q10 improved oocyte quality in older women [297]. As for young women with diminished ovarian reserves, coenzyme Q10 supplementation also improves oocyte quality and embryo quality [298]. To date, various pharmaceuticals have been studied for their potential effects on somatic aging and ovarian aging. However, the application of anti-aging drugs remains limited, requiring further research into optimal dosages and timing for effective treatment.

## 4. Discussion

With the accelerated aging of the global population, it has become increasingly imperative to investigate the underlying factors of aging and potential intervention strategies. Among all organs in the female body, the ovaries are the first to exhibit signs of aging. While traditionally associated with menopause and infertility, ovarian aging has far-reaching implications due to the widespread presence of ERs throughout the body. These issues encompass declines in multi-system and multi-organ functions, which may ultimately impact women's overall health and well-being. The risks associated with ovarian aging have not received adequate attention, and research in this area remains relatively sporadic. This review provides a comprehensive analysis of the impact of ovarian aging on somatic age-related diseases and evaluates therapeutic interventions aimed at mitigating both ovarian and systemic aging.

Ovarian aging is intrinsically intertwined with the broader process of organic aging. Accumulating evidence indicates a significant correlation between ovarian aging and a spectrum of somatic aging-related diseases. While previous research primarily focused on the increased risks of cardiovascular disease, osteoporosis, and AD in postmenopausal women, our review highlights the broader impact of ovarian aging on multiple systems, such as urinary, respiratory, and digestive systems. Ovarian aging induces both functional and structural changes within the body's systems and organs, thereby contributing to age-related diseases in multiple organs. Hormonal fluctuations during ovarian aging alter the cellular microenvironment, increasing susceptibility to age-related diseases. The structural modifications and functional decline in organs such as the kidneys, bladder, liver, and lungs, contribute to the pathogenesis of age-related diseases such as chronic kidney disease, chronic obstructive pulmonary disease, idiopathic pulmonary fibrosis, non-alcoholic fatty liver disease, and other related conditions. The mechanisms as well as the structural and functional changes related to ovarian aging and their impact on age-related diseases require further investigation. Chronic low-grade inflammation, often accompanying ovarian aging, may accelerate the aging process at the organismal level. Future research should explore the potential of anti-inflammatory strategies to enhance ovarian function and their implications for systemic health.

Racial and ethnic differences significantly influence both the progression of ovarian aging and its impact on age-related diseases. Racial and ethnic background may play an important role in determining the lifespan of ovarian function, as evidenced by the differing incidence rates of premature menopause among White, Black, and other demographic groups [299]. Additionally, AMH, an indicator of ovarian function, shows a more pronounced age-related decline in African-American and Latina women compared to White women, potentially due to genetic variants associated with AMH [299]. Furthermore, a multiracial cohort study of 951 healthy women aged 25-45 years revealed higher AMH levels in White women compared to Latina women [300]. Ovarian aging also exerts variable impacts on age-related diseases across different demographic groups. A cross-sectional study involving 308 women revealed that a significantly higher proportion of African American women reported experiencing physical symptoms such as hot flashes, dizziness, urinary leakage, and vaginal dryness compared to White women (46% vs. 30%, *p* < 0.001) [301]. Possibly due to the partially attenuated effects of AMH on HDL and hypertension, African American and Latina women are 2 to 9 times more likely than White women to have poorer cardiovascular metabolic outcomes—measured by HDL, waist circumference, and hypertension [300]. Longitudinal data also reveal that cardiovascular risk increases among overweight and obese Black women but not in their White counterparts [302]. Additionally, a cross-sectional study conducted from 2005 to 2018, encompassing 4,012 postmenopausal women in America, revealed that ovarian aging had varying impacts on osteoporosis across different populations [303]. In the non-Hispanic White cohort, the prevalence of osteoporosis and osteopenia, as determined by BMD assessments, was found to be 9.9% and 54.14%, respectively [303]. In contrast, the non-Hispanic Black cohort reported lower prevalence rates of osteoporosis at 3.8% and osteopenia at 28.85% [303]. At the age of 65, the prevalence of pelvic prolapse was observed to be 4.2% for Japanese, 4.8% for Chinese, 8.9% for black, 9.7% for white, and 33.9% for Hispanic women [304]. When compared to White women, the HRs for pelvic prolapse were 3.09 (95% CI = 2.18-4.39) for Hispanic women, 0.96 (0.71-1.31) for Black women, 0.43 (0.22-0.85) for Chinese women, and 0.48 (0.26-0.88) for Japanese women [304]. Overall, ovarian aging and its impact on age-related diseases show racial and ethnic disparities.

For age-related disorders associated with ovarian aging, early hormone therapy in the menopausal transition has demonstrated efficacy in reducing the frequency and severity of hot flashes, mitigating cardiovascular risks, preventing bone loss and osteoporotic fractures, and alleviating vaginal atrophy [305]. Regarding non-hormonal novel therapies, glucagon-like peptide-1 (GLP-1) agonists have the potential to reduce the risk of cardiovascular disease associated with ovarian aging during the menopausal transition [305]. Emerging therapies, including fazolinolide, a neurokinin 3 receptor (NK3) antagonist, effectively alleviate vasomotor symptoms (VMS- hot flashes, night sweats) by reducing their severity by up to 90%, presenting a potential alternative to estrogen-based treatments [306]. Independent of pharmacological interventions, adherence to a nutrient-rich, plant-based diet, such as the Mediterranean diet, and regular physical activity are associated with reduced biological aging and improved systemic health [307-309]. Supplementation with vitamins D and K prevents bone loss, improves immune function, and reduces cardiovascular risk, thereby promoting healthy aging and well-being [310].

Our study provides a detailed and comprehensive review of the impact of ovarian aging on age-related diseases and effective anti-aging drugs. Ovarian aging may drive the onset of somatic aging and various age-related diseases. Some agents including metformin, nicotinamide mononucleotide, rapamycin, and resveratrol, have been identified as potential candidates for anti-aging therapy, however, their safety and efficacy require further investigation. Raising awareness about ovarian aging and its potential implications, particularly among younger women, may facilitate early intervention and health management, ultimately improving long-term health outcomes and quality of life. Considering the impacts of ovarian aging on the physical and mental health of older women, it is imperative to implement policies or programs, such as educational lectures and preventive measures, within community and healthcare settings to mitigate its negative impacts. Although various treatment options for addressing ovarian aging and age-related diseases have been summarized, further research is necessary to gather additional data and determine the most effective therapeutic strategies. More longitudinal studies are warranted to gain a deeper understanding of the long-term impacts of ovarian aging on women's health, as well as to evaluate the long-term effectiveness of various interventions. Additional clinical trials are needed to evaluate the efficacy and safety of interventions for ovarian aging in postmenopausal women.
